# Commentary: Tumor associated neutrophils promote prostate cancer progression by mediating neutrophil trap secretion through PSMA1- NF-κB-HIF-1α signaling axis

**DOI:** 10.3389/fimmu.2026.1932868

**Published:** 2026-08-27

**Authors:** Yiling Qin, Zijun Wang, Wujie Chen, Qingyu Wan, Mingxia Ding, Yidao Liu, Shunxin Lu, Jiyao Yang, Yinglong Huang, Haihao Li

**Affiliations:** 1 Department of Urology, The Second Affiliated Hospital of Kunming Medical University, Kunming, Yunnan, China; 2 Department of Urology, The First People's Hospital of Yunnan Province, Kunming, China; 3 Department of Urology, Dehong Prefecture People`s Hospital, Mangshi, Yunnan, China; 4 Department of Urology, Lincang Traditional Chinese Medicine Hospital, Lincang, Yunnan, China

**Keywords:** immunotherapy, neoadjuvant hormone therapy, prostate cancer, tertiary lymphoid structures, tumor microenvironment

## 1 Introduction

What role do neutrophils play in the tumor microenvironment? A recent study by Dai and colleagues offers a compelling answer (1). This is the first study to systematically elucidate the molecular mechanism by which tumor-associated neutrophils (TANs) drive neutrophil extracellular trap (NET) formation through the PSMA1-NF-κB-HIF-1α signaling axis, thereby promoting malignant progression in prostate cancer (PCa). In parallel, the authors constructed a 6-gene prognostic model based on NET-related genes, providing a novel tool for risk stratification and individualized therapy in PCa. Integrating retrospective clinical analysis, multi-database mining, machine learning modeling, single-cell sequencing, and organoid-based functional validation, this study establishes a complete and rigorous chain of evidence. We hold great admiration for the academic contributions of the research team. At the same time, we offer several constructive suggestions regarding aspects that merit further exploration, hoping to provide reference for future investigations.

## 2 Major academic contributions

### 2.1 First extension of NET-related prognostic models to prostate cancer

NET-related gene prognostic models have been reported in various solid tumors, including breast cancer, lung cancer, and gastric cancer (2–5). However, the prognostic value of NETs in prostate cancer has lacked systematic investigation. The present study fills this gap. By intersecting 16,045 differentially expressed genes with NET-related gene sets, the authors identified 314 NET-DEGs, and further screened six key genes (PLCG1, PLCB2, AGER, ALDOA, FCGR2B, and PSMA1) through univariate Cox regression and LASSO regression to construct a risk model. This model achieved AUCs of 0.78, 0.80, and 0.84 for predicting 1-, 3-, and 5-year biochemical recurrence in the training set, with robust performance maintained in the validation cohort. Notably, these findings corroborate those of Zheng et al. (6), who similarly demonstrated that NET-related molecular signatures effectively distinguish prognostic differences in prostate cancer patients. Building upon this foundation, the present study further reinforces model reliability through multi-dimensional validation, including independent cohorts, single-cell localization, and organoid-based functional experiments.

### 2.2 Precise single-cell localization of PSMA1^+^ TAN subpopulations

Single-cell sequencing analysis represents a major highlight of this study. Through single-cell transcriptomic analysis of 32 samples (14 normal and 18 tumor), the authors identified 10 neutrophil subclusters, four of which were classified as TANs. Critically, the six key genes in the risk model were precisely localized to two TAN subclusters (Neu_c09_HLA and Neu_c07_KLK3), among which PSMA1 exhibited the highest expression in Neu_c09_HLA—a subcluster enriched for HIF-1α and NF-κB signaling pathways. This finding provides a spatial dimension for understanding the function of NET-related genes within specific cellular subsets, and identifies cellular-level intervention nodes for subsequent targeted therapy. This “risk gene → cell subcluster → signaling pathway” stepwise localization strategy carries significant methodological implications.

### 2.3 Organoid models provide direct functional evidence for the pro-tumor role of NETs

Unlike most prognostic model studies that remain at the bioinformatics level, the present study performed functional validation using patient-derived prostate cancer organoids. The authors established three human PCa organoids and demonstrated, through two co-culture approaches (conditioned medium and direct co-culture), that PSMA1-knockdown HL-60 cells significantly inhibited organoid growth. Concurrently, PSMA1 knockdown reduced the expression of NET markers MPO and CitH3, suggesting that PSMA1 is directly involved in NET formation. This validation gradient—”from gene signature to cellular function to organoid phenotype”—substantially strengthens the rigor of causal inference. In recent years, organoid models have gained increasing attention in prostate cancer immune microenvironment research. Liu et al. (7) utilized organoid-CRISPR-Cas9 to construct an orthotopic immunocompetent mouse model for studying tertiary lymphoid structures, while Harris et al. (8) employed organoids to evaluate the anti-tumor activity of CAR-neutrophils. The present study further expands the application of organoids in assessing immune cell–tumor interactions.

## 3 Aspects meriting further exploration

### 3.1 The drivers of PSMA1 upregulation in neutrophils remain unclear

The present study demonstrates that PCa cells induce PSMA1 upregulation in neutrophils; however, the specific molecular drivers remain to be elucidated. Which factors in the tumor microenvironment mediate this effect? Candidate molecules include tumor-secreted IL-8—which, as demonstrated by Luo and Chen (9), can be upregulated by NETs themselves, forming a positive feedback loop—as well as GM-CSF, TGF-β, and metabolic products such as lactate. Furthermore, does PSMA1 upregulation occur through transcriptional activation, protein stabilization, or other mechanisms? To address these questions, we propose that future studies systematically screen for key factors in PCa-conditioned medium, employ neutralizing antibodies or genetic knockdown approaches to validate their contribution to PSMA1 upregulation, and integrate transcriptomic and proteomic analyses to dissect the underlying molecular mechanisms.

### 3.2 The molecular network of PSMA1-mediated NET formation requires further dissection

As a catalytic subunit of the 20S proteasome core particle, PSMA1 is theoretically involved in global protein degradation (10). However, the present study reveals its specific regulation of NET formation through the NF-κB-HIF-1α axis, and the mechanistic basis of this pathway specificity warrants in-depth investigation, which may inform future therapeutic development. Does PSMA1 activate NF-κB through degradation of specific inhibitory factors, such as IκBα? This question could be addressed in future studies by employing proteomics to identify the substrate repertoire regulated by PSMA1. Whether HIF-1α activation represents a downstream event of NF-κB signaling or constitutes an independent pathway could be addressed through ChIP-seq or CUT&Tag analysis to map the genome-wide binding sites of NF-κB and HIF-1α. Furthermore, to elucidate how the core effector molecules of NET formation, such as MPO and CitH3, are regulated at the transcriptional or post-transcriptional level, specific inhibitors could be employed to achieve stepwise blockade of pathway nodes, thereby dissecting the hierarchical regulatory relationships within the signaling cascade. To facilitate such mechanistic investigations, we have compiled a panel of pharmacological agents targeting key nodes in this signaling axis (**Table 1**). Systematic application of these inhibitors across distinct prostate cancer models would not only help establish the hierarchical architecture linking PSMA1, NF-κB, HIF-1α, and NET formation, but may also identify the most therapeutically accessible intervention points within this regulatory network.

**Table 1:** Pharmacological inhibitors for dissecting the PSMA1-NF-κB-HIF-1α-NET signaling axis.

Target node	Inhibitor	Mechanism of action	Key evidence/application	Ref(s)
Proteasome (PSMA1)	MG132	Reversible peptide aldehyde proteasome inhibitor; blocks IκBα degradation and NF-κB activation	Used in prostate cancer cells to assess proteasome-dependent AR regulation; inhibits IκB degradation	(17)
Proteasome (PSMA1)	Bortezomib	Reversible boronic acid dipeptide; FDA-approved 20S proteasome inhibitor	Validated in prostate cancer models; antagonized by EGCG via autophagic mechanism	(17)
NF-κB pathway	BAY 11-7082	Irreversible IκBα phosphorylation inhibitor; prevents NF-κB nuclear translocation	Directly validated to abrogate NET formation in human neutrophils and mouse peritonitis models	(18)
NF-κB pathway	Ro 106-9920	NF-κB inhibitor; structurally distinct from BAY 11-7082	Directly validated to suppress NETosis via reduction of NF-κB p65 phosphorylation	(18)
NF-κB pathway	SC75741	NF-κB nuclear translocation inhibitor	Blocks NF-κB-driven transcription; can be used to verify the necessity of NF-κB in PSMA1-mediated NET formation	—
HIF-1α pathway	PX-478	Inhibits HIF-1α transcription, translation, and deubiquitination	Directly validated to restore NETosis and ROS production in HIF-1α-dependent neutrophil suppression models	(19)
HIF-1α pathway	LW6	HIF-1α inhibitor; promotes HIF-1α degradation	Directly validated to reverse hypoxia-induced NET suppression in neutrophil-biofilm co-culture	(19)
HIF-1α pathway	Echinomycin	Inhibits HIF-1α/DNA binding; blocks HIF-1α transcriptional activity	Prevents HIF-1α-driven transcription; validated in multiple tumor models	(19)
NETosis (PAD4)	GSK484	Reversible, selective PAD4 inhibitor	Directly validated to inhibit neutrophil NETosis; reduces lung metastasis in breast cancer models	(20, 21)
NETosis (PAD4)	Cl-amidine	Small-molecule pan-PAD inhibitor (PAD1-4)	Directly validated to block NETosis via inhibition of histone citrullination; reduces inflammation and metastasis in multiple disease models	(21)
Neutrophil Elastase	Sivelestat	Selective neutrophil elastase (NE) inhibitor	Directly validated to inhibit NE-mediated NET formation; suppresses NET-driven tumor progression	(21)
Neutrophil Elastase	GW311616A	Potent, specific neutrophil elastase inhibitor	Directly validated to inhibit NE activity and NET formation; more effective than Sivelestat in certain contexts	(21)

SC75741, while well-established as an NF-κB nuclear translocation inhibitor in multiple cell systems, awaits direct validation in the context of NET regulation.

### 3.3 The functional heterogeneity of NETs in prostate cancer warrants attention

Neutrophils exhibit high functional plasticity in tumors, capable of assuming either an N1 anti-tumor phenotype or an N2 pro-tumor phenotype. The present study predominantly supports the pro-tumor role of NETs; however, whether NETs exert anti-tumor effects under specific contexts remains incompletely elucidated. Previous studies have demonstrated that neutrophil elastase (NE) can degrade the tumor stroma to inhibit metastasis (11), whereas the present study suggests that NE may promote progression through NETs. Furthermore, Harris et al. (8) showed that CAR-engineered neutrophils can directly kill tumor cells. This functional heterogeneity implies that simply categorizing “NETs” as pro-tumor factors may be overly reductive. In future investigations, we propose that NETs be classified into functional subtypes based on their compositional features (protein profiles, nucleic acid structures), inducing stimuli (hypoxia, cytokines, tumor contact), and microenvironmental context (inflammatory status, immune cell composition), in order to elucidate the differential impacts of distinct NET subtypes on tumor progression and immunotherapy response, thereby providing a deeper understanding of these complex biological processes.

## 4 Paradigm reflections and future perspectives

Most bioinformatics-based prognostic model studies stop at model construction and immune infiltration analysis. The distinctive value of this study lies in its completion of the closed loop from model to mechanism—not only statistically validating the model’s efficacy (AUC, independent prognostic factors, nomogram), but also elucidating the functional mechanism of PSMA1, the core gene in the model, through single-cell localization and organoid-based experiments. This “bioinformatics-driven hypothesis → experimental mechanism validation” reverse biology paradigm parallels the approach of Jia et al. (12) in their PCP4 study—both begin with big data screening and ultimately return to molecular mechanisms and functional validation in animal and organoid models. The advantage of this paradigm is its efficiency and clear targeting; however, a potential risk is the possible neglect of molecules that, while not reaching statistical significance in the initial screening, may hold substantial biological importance—a caveat that warrants particular attention.

The pro-tumor mechanism of NETs revealed in this study engages in an interesting dialogue with other immune microenvironment studies in prostate cancer. Ban et al. (13) found that IL-33 recruits lymphocytes through the ST2/NF-κB pathway to exert anti-tumor effects, whereas NETs may conversely limit immune cell infiltration through physical barriers or inhibition of T cell function. These two forces appear to constitute a “pro-immune” versus “anti-immune” tug-of-war within the prostate cancer microenvironment. Liu et al. (7) demonstrated the prognostic value of tertiary lymphoid structures (TLS) in prostate cancer and the promoting effect of neoadjuvant hormone therapy on their formation—TLS as structural foundations of immune activation stand in stark contrast to NETs as functional mediators of immune suppression. This “structure-function” dialectic suggests that future studies should investigate whether NETs influence TLS formation and function, and whether targeting NETs could unleash the anti-tumor potential of TLS, indicating that this line of research holds considerable promise.

Notably, prostate cancer is not a single disease entity but comprises multiple subtypes with distinct cellular origins and genomic drivers, including adenocarcinoma (luminal origin), neuroendocrine prostate cancer (NEPC, often arising from luminal cells under therapeutic pressure), and ductal adenocarcinoma (14). However, whether NETs derived from tumors of different cellular origins exhibit distinct functional properties remains entirely unexplored. Given that TANs are educated by the tumor microenvironment, it is plausible that the composition and function of NETs differ between AR-driven luminal tumors and AR-independent NEPC, which may partly explain the differential immunotherapy responses observed across PCa subtypes (15, 16). Future studies should address this question by comparing NET profiles in preclinical models of distinct PCa subtypes—such as Pten-loss versus Rb1/Trp53-loss models—and by performing subtype-stratified analyses in clinical cohorts. Such investigations could reveal whether NET-targeting strategies need to be tailored to specific PCa subtypes for optimal therapeutic benefit.

## 5 Conclusion

In summary, Dai and colleagues are the first to introduce NET-related prognostic models into prostate cancer research, and to innovatively reveal the central role of the PSMA1-NF-κB-HIF-1α signaling axis in TAN-mediated NET formation and tumor progression. The significance of this study lies not only in providing a multi-dimensionally validated prognostic tool, but also in elevating NETs from a “correlative phenomenon” to a causal “driving factor.” Our suggestions—elucidating the upstream regulatory network of PSMA1, dissecting the molecular details of NET formation, and attending to the functional heterogeneity of NETs—are offered with the aim of providing reference points for the continued deepening of this research direction. We look forward to further breakthrough discoveries from the research team in the interplay between NETs and prostate cancer.
